# N-Acetylcysteine Attenuates Oxidative Stress and Preserves Red Blood Cell Quality During Whole Blood Storage

**DOI:** 10.3390/antiox15070858

**Published:** 2026-07-08

**Authors:** Sonia Eligini, Lisa Brocca, Alice Mallia, Arianna Valeriano, Erica Gianazza, Cristina Banfi

**Affiliations:** 1 Unit of Functional Proteomics, Metabolomics and Network Analysis, Centro Cardiologico Monzino IRCCS, 20138 Milan, Italy; sonia.eligini@cardiologicomonzino.it (S.E.); lisa.brocca@cardiologicomonzino.it (L.B.); alice.mallia@cardiologicomonzino.it (A.M.); erica.gianazza@cardiologicomonzino.it (E.G.); 2 Laboratory of Medical Genetics, Cytogenetics and Molecular Genetics, European Institute of Oncology IEO, IRCCS, 20141 Milan, Italy; arianna.valeriano@ieo.it

**Keywords:** whole blood, red blood cells, N-acetylcysteine, albumin, free hemoglobin, proteomics

## Abstract

Whole blood (WB) storage induces biochemical and biomechanical alterations that may compromise red blood cell (RBC) quality. Since oxidative stress is a major driver of storage lesions, we investigated whether N-acetylcysteine (NAC) could attenuate these changes during refrigerated storage. WB from healthy donors was stored at 4 °C for 42 days with or without NAC, added either once at baseline or every 10 days. Plasma albumin proteoforms were assessed by liquid chromatography–mass spectrometry, free hemoglobin species by spectrophotometry, plasma proteomic changes by proximity extension assay, and RBC hemorheological properties by LORRCA analysis. Storage decreased reduced albumin (HSA-SH) and increased oxidized albumin (HSA-Cys), indicating plasma oxidation. Free oxyhemoglobin, deoxyhemoglobin, and methemoglobin increased, consistent with hemoglobin oxidation and hemolysis. Storage also induced plasma proteomic alterations and impaired RBC osmotic and deformability parameters. NAC preserved albumin redox status, limited free hemoglobin accumulation, and attenuated storage-induced proteomic changes. Moreover, NAC partially preserved RBC osmotic and rheological properties, particularly parameters related to osmotic fragility and hydration. No clear advantage of 20 mM over 10 mM NAC was observed. Overall, NAC attenuated oxidative and functional alterations associated with refrigerated whole blood storage, supporting further investigation of antioxidant supplementation as a strategy to mitigate storage lesions under ex vivo conditions.

## 1. Introduction

Whole blood was long considered the transfusion product for a broad range of clinical indications [1]. However, since the introduction of blood component therapy in the mid-1970s, its use has markedly declined, and most blood banks now separate the whole blood units into red blood cells (RBCs), platelets, and plasma, in order to transfuse only the component required by the patient. This strategy offers several advantages, including administering only the necessary component, the possibility of treating multiple recipients from a single donation, reduced waste and costs, and greater flexibility in storage. Nevertheless, component therapy may be less effective in the setting of massive hemorrhage, where the rapid restoration of blood volume and oxygen-carrying capacity is required [2,3]. In this context, several studies have highlighted the high prevalence of coagulopathy in trauma patients undergoing massive transfusion with blood components [4,5,6]. Moreover, transfusion with RBCs, plasma, and platelets in a 1:1:1 ratio may lead to dilutional coagulopathy because of the relatively low content of platelets and coagulation factors compared with whole blood [7]. Together with easier administration and a lower risk of critical transfusion errors, this limitation has renewed interest in the clinical use of whole blood [8]. Consistently, recent studies have shown that whole blood transfusion is associated with improved survival in patients with hemorrhagic shock and severe trauma compared with component-based resuscitation using RBCs, plasma, and platelets [9,10,11].

Despite these advantages, cold storage induces a series of structural, biochemical, and metabolic alterations in blood components, collectively referred to as storage lesions [12,13]. Among the mechanisms involved, oxidative stress plays a pivotal role. RBCs are extremely susceptible to oxidative damage because of the high content of polyunsaturated fatty acids in their membrane and the abundance of oxidation-sensitive biomolecules [14,15]. Oxidative stress leads to the generation of reactive species that damage structural proteins, enzymes, and soluble mediators. Because cellular alterations are reflected in the extracellular milieu, changes occurring in blood cells during storage also affect plasma composition. 

The most abundant plasma protein, albumin (HSA), is characterized by several activities, including antioxidant activity [16,17]. Depending on its redox state, HSA exists in three major proteoforms: mercaptoalbumin (HSA-SH), the reduced proteoform that exhibits marked antioxidant activity in the plasma compartment; thiolated albumin (HSA-Cys), in which the free thiol group forms a reversible disulfide bond with low-molecular weight thiols; and non-mercaptoalbumin-2, in which the thiol group is irreversibly oxidized to sulphinic or sulphonic acid [18,19]. Plasma HAS-SH is able to neutralize both reactive oxygen species and free radicals, thereby reducing oxidative stress in cells and tissues [20]. However, this process results in its conversion to oxidized forms [21]. Thus, shifts in HSA proteoforms may provide a useful readout of oxidative changes occurring during blood storage.

RBCs are also equipped with several antioxidant systems; however, prolonged storage progressively weakens these defenses and promotes oxidative damage, particularly at the level of hemoglobin (Hb), leading to the formation of methemoglobin and superoxide anions (O_2_^·−^) [22]. In addition, low temperature promotes methemoglobin denaturation, with the consequent release of free hemin and formation of hemichromes, which bind to Band 3, the most abundant integral membrane protein of the RBC membrane. This process promotes Band 3 clustering, membrane destabilization, and progressive hemolysis [23,24,25]. Alterations in RBC redox homeostasis during storage, together with the increased hemolysis and the release of pro-oxidant molecules, may therefore contribute to systemic oxidative imbalance after transfusion.

Several strategies have been explored to limit oxidative damage and mitigate storage lesions, including the supplementation with antioxidant molecules, anaerobic storage, and the scavenging of harmful molecules [26]. Because endogenous antioxidant defenses are progressively consumed during storage, exogenous antioxidants such as ascorbic acid, vitamin E, and N-acetylcysteine (NAC) have been proposed as additives to improve RBC preservation. In fact, it has been shown that supplementation with ascorbic acid, either alone or in combination with uric acid, reduces oxidative stress and enhances antioxidant defenses, thereby preserving or producing glutathione [27,28,29]. Similarly, beneficial effects have been described with membrane-interacting antioxidants including vitamin E analogs [30]. NAC is another important antioxidant and its effect has been examined both alone and in combination [31,32]. NAC is a particularly attractive candidate, as it has been shown to reduce hemolysis and partially restore the capacity of stored RBCs to metabolize H_2_O_2_ [31].

In the present study, we investigated whether NAC improves the quality of whole blood stored under refrigerated conditions. To this end, we assessed RBC rheological properties using the state-of-the-art laser-assisted optical rotational cell analyzer (LORRCA MaxSis), evaluated the antioxidant status of plasma by measuring HSA proteoforms, and characterized plasma proteomic changes occurring during storage in the absence or presence of NAC. This integrated approach was designed to identify molecular and functional markers of oxidative injury and to explore whether NAC may represent a useful strategy for optimizing whole blood storage conditions.

## 2. Materials and Methods

### 2.1. Sample Collection

Blood was drawn from the antecubital vein of healthy subjects (n = 18; age 34.22 ± 9.19, mean and SD) who had not taken any drugs during the previous 10 days. Samples were collected into tubes containing CPD solution (129 mM dextrose, 105 mM citrate, and 16 mM phosphate; Sigma-Aldrich, Merck Life Science S.r.l., Milan, Italy) as anticoagulant, using an anticoagulant-to-whole blood ratio of 1:14. Whole blood was then aliquoted into separate tubes and stored under static conditions at 4 °C for up to 42 days in the presence or absence of NAC (10 or 20 mM; Sigma-Aldrich, Merck Life Science S.r.l., Milan, Italy). Two storage protocols were performed: (i) whole blood storage for 42 days with a single addition of NAC (10 or 20 mM) at baseline (T0); and (ii) whole blood storage for 42 days with NAC (10 or 20 mM) added at T0 and then every 10 days.

The study was conducted according to the guidelines of the Declaration of Helsinki and approved by the Ethics Committee of Centro Cardiologico Monzino IRCCS (R1537/21-CCM1626). Written informed consent was obtained from all subjects involved in the study.

### 2.2. Hematological and Rheological Measurements

Hematological parameters were recorded using Sysmex XN450 (Sysmex Italia S.R.L., Milan, Italy).

Rheological parameters were assessed using the Laser-assisted Optical Rotational Cell Analyzer (LORRCA MaxSis; RR Mechatronics Manufacturing B.V., Zwaag, The Netherlands). Before analysis, blood samples, either collected immediately after donation (T0) or after 42 days of storage (T42), were diluted in 5 mL of isotonic solution Elon ISO (RR Mechatronics Manufacturing B.V., Zwaag, The Netherlands). For osmoscan analysis and deformability analysis, suspensions containing 1000 × 10^6^ RBC and 100 × 10^6^ RBC, respectively, were prepared and gently mixed. For osmoscan analysis, RBC deformability was measured over a continuous osmotic gradient ranging from 0 to 500 mOsmol/Kg at a constant shear stress of 30 Pa. The following parameters were recorded:

- Minimum elongation index (EI min): the minimum deformability of RBC at hypotonic osmolality measured by the elongation index;
- Maximum elongation index (EI max): the maximal deformability of RBC at isotonic osmolality measured by the elongation index;
- Osmolality at minimum of EI (O min): value of the hypotonic osmolality where 50% of RBC are hemolyzed;
- Osmolality at half of maximal RBC elongation (O hyper): value of the hypertonic osmolality where 50% of the EI max is achieved;
- Osmolality at maximum elongation index (O EI max): value of osmolality where EI max is achieved;
- Area under the curve (AUC): the area between the starting point in the hypo-osmolar region (O min) and the ending point in the hyper-osmolar region (500 mOsm/Kg).

RBC deformability under dynamic flow conditions was also evaluated using the deformability mode of the LORRCA. Whole blood was exposed to 9 increasing shear stress values (0.30; 0.53; 0.95; 1.69; 3.00; 5.33; 9.49; 16.87; 30.00 Pa) and the following parameters were obtained:

- EI max: the maximum extent to which cells can stretch;
- SS½: shear stress which induces shape change at half of the maximum elongation; index (EI max) and is calculated by the Lineweaver–Burk analysis.

### 2.3. Detection of Plasma Albumin Proteoforms by Liquid Chromatography Mass Spectrometry

The relative abundance of HSA proteoforms was determined by LC–mass spectrometry using a Xevo TQ-S micro triple-quadrupole mass spectrometer coupled to an ACQUITY UPLC M-Class system (Waters Corporation, Milford, CT, USA), according to a previously described method with minor adaptations [33].

Briefly, plasma samples were diluted 1:400 in 30% acetonitrile containing 0.1% formic acid and centrifuged at 14,000× *g* for 10 min at 4 °C. Aliquots of 2 µL were injected onto an ACQUITY UPLC Protein BEH C4 column (300 Angstrom, 1.7 µm, 1.0 × 50 mm; Waters Corporation, Milford, MA, USA). Chromatographic separation was performed at a flow rate of 5 µL/min with the column maintained at 40 °C. Mobile phases consisted of water/0.1% formic acid (solvent A) and acetonitrile/0.1% formic acid (solvent B), and the elution gradient was applied as previously reported [34].

Mass spectra were acquired over 4 min in positive electrospray ionization mode in the *m*/*z* range 1100–1350. Instrument settings were as follows: scan time, 1 s; capillary voltage, 3 kV; cone voltage, 90 V; desolvation temperature, 350 °C; and source temperature, 150 °C. Spectral deconvolution was performed with the MaxEnt1 function implemented in MassLynx version 4.0 (Waters Corporation, Milford, CT, USA), using a mass range of 40,000–80,000 Da, a uniform Gaussian model with 1.1 Da peak width at half height, minimum adjacent peak intensity ratios of 33% on both sides, and iterative convergence.

The relative abundance of HSA proteoforms was calculated from the signal intensities of HSA-SH and the oxidized proteoform HSA-Cys (+120 ± 2 Da), as previously described [33]. Protein mass was assigned after MaxEnt1 deconvolution on the basis of the centroid of the mass distribution rather than the monoisotopic mass. Across all analyzed samples, the coefficient of variation for HSA-Cys quantification was 7.0 ± 1.1% (mean ± SEM).

### 2.4. Determination of Plasma Free Hemoglobin, Methemoglobin, Oxygenated Hemoglobin, and Hemolysis

The concentrations of the different hemoglobin species in plasma were determined by absorbance measured using a NanoDrop One Spectrophotometer (Thermo Fisher Scientific Inc., Wilmington, DE, USA). Oxyhemoglobin, which contains bound oxygen, exhibits a characteristic absorbance peak at 414 nm; deoxyhemoglobin, which lacks bound oxygen, shows a peak at 431 nm; and methemoglobin, which is unable to bind oxygen, displays a peak at 406 nm [35,36]. The concentrations of the three forms of hemoglobin were calculated using the corresponding extinction coefficients and molecular weights, namely 524,280 M^−1^ cm^−1^ and 64.5 kDa for oxyhemoglobin; 552,160 M^−1^ cm^−1^ and 64.5 kDa for deoxyhemoglobin; and 167,000 M^−1^ cm^−1^ and 68.0 kDa for methemoglobin [37].

The percent of hemolysis has been calculated using the following formula:

$$\mathrm{Percent}\;\mathrm{hemolysis}\;(\%)\;=\;\frac{(100-Hematocrit)\times free\;hemoglobin\;in\;plasma}{Total\;hemoglobin}$$

### 2.5. Determination of Plasma Proteome Profile

The plasma proteome profile at T0 and T42 was assessed using the Proximity Extension Assay (PEA) technology with the Olink Target 96 (Olink Proteomics, Uppsala, Sweden). This assay is based on pairs of oligonucleotide-labeled antibodies that bind simultaneously to the target protein. Upon dual recognition of the analyte, the two DNA barcodes come into proximity and generate a unique amplifiable sequence, which is subsequently detected by microfluidic real-time PCR. Threshold cycle (Ct) values derived from internal and external controls were subjected to quality control and normalization according to the manufacturer’s procedure. The list of proteins included in the Olink panel is provided in Supplemental Table S1. Protein abundance was expressed as Normalized Protein eXpression (NPX) values on a log_2_ scale. Samples that did not meet the quality control criteria were excluded from the analysis. Results are reported as median NPX values with interquartile ranges.

### 2.6. Protein Interaction Analysis

A protein–protein network was constructed using data from the STRING database (https://string-db.org/).

### 2.7. Statistical Analysis

Data were analyzed using GraphPad Prism software version 10. Statistical analysis between the two groups was performed using paired Student’s *t*-test. Comparisons among three or more groups were performed with one-way analysis of variance (ANOVA) in association with multiple comparison post hoc tests. A *p*-value < 0.05 was considered statistically significant.

## 3. Results

### 3.1. Prolonged Whole Blood Storage Decreases the Reduced Albumin and Increases the Oxidized Proteoform

**Figure 1:**
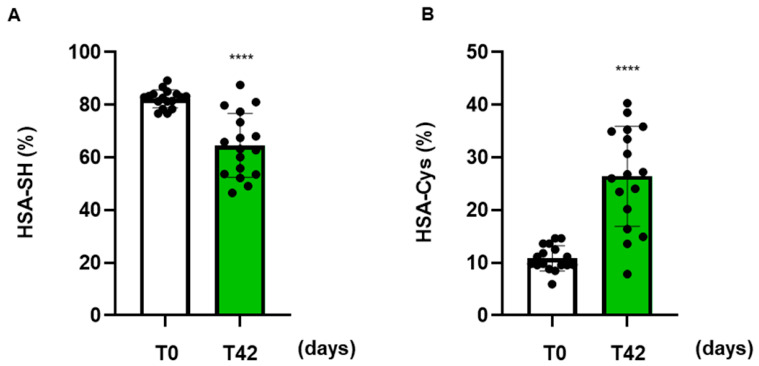
Storage-dependent changes in plasma albumin proteoforms. The levels of the HSA proteoforms were measured in plasma by mass spectrometry at T0 (white) and after 42 days of whole blood storage at 4 °C (T42, green). (**A**) Plasma levels of mercaptoalbumin HSA-SH; n = 17. (**B**) Plasma levels of the thiolated proteoform HSA-Cys; n = 17. **** *p* < 0.0001.

Prolonged storage of whole blood was associated with a shift in plasma HSA redox status, consistent with increased oxidative stress. After 42 days of storage (T42), the relative abundance of HSA-SH, the reduced form of albumin, was significantly lower than in fresh samples collected at baseline (T0) (Figure 1A). Conversely, the relative abundance of HSA-Cys was significantly increased at T42 (Figure 1B). Taken together, these results support the occurrence of progressive oxidative remodeling of plasma HSA during whole blood storage.

### 3.2. Whole Blood Storage Promotes Hemoglobin Oxidation and Plasma Accumulation of Free Hemoglobin Species

**Figure 2:**
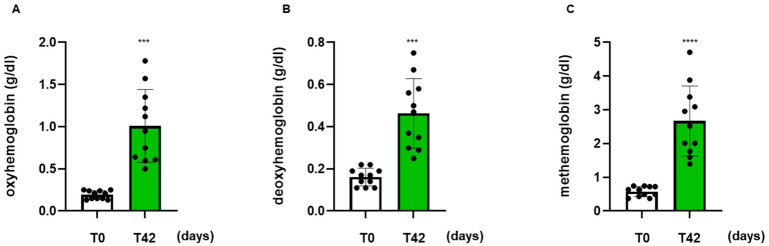
Storage-dependent increase in plasma free hemoglobin species. Plasma levels of oxyhemoglobin, deoxyhemoglobin, and methemoglobin were measured by a NanoDrop spectrophotometer at T0 (white) and after 42 days of whole blood storage at 4 °C (T42, green). (**A**) plasma oxyhemoglobin levels; n = 11. (**B**) plasma deoxyhemoglobin levels; n = 11; (**C**) plasma methemoglobin levels; n = 11; *** *p* < 0.001; **** *p* < 0.0001.

After 42 days of whole blood storage, a marked increase in RBC hemolysis was detected (0.99% ± 0.22 at T0 and 4.96% ± 1.97 at T42) and plasma levels of free hemoglobin species were significantly increased compared with baseline (T0). Indeed, as shown in Figure 2A–C, oxyhemoglobin, deoxyhemoglobin, and methemoglobin were all markedly elevated at T42. These findings indicate that prolonged whole blood storage is associated with hemoglobin oxidation and increased release of hemoglobin into the plasma compartment.

### 3.3. Whole Blood Storage Alters the Plasma Proteomic Profile

Whole blood storage was associated with a marked remodeling of the plasma proteomic profile. As shown in Supplemental Table S2, comparison of samples collected at baseline (T0) and after storage (T42) revealed an overall increase in the abundance of most proteins detected by PEA. In contrast, only three proteins, leukocyte immunoglobulin-like receptor subfamily B member 4 (LILRB4), integral membrane protein 2A (ITM2A), and stanniocalcin-1 (STC1), showed a significant decrease after storage. Overall, these findings suggest that whole blood storage alters the plasma protein milieu, with a tendency toward increased abundance of several immune- and stress-related mediators.

**Figure 3:**
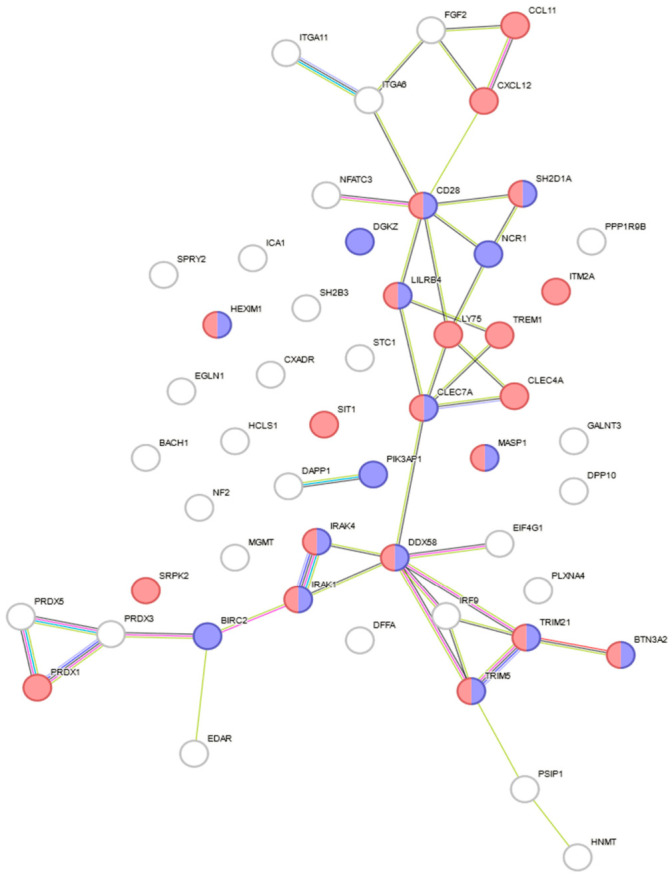
STRING network analysis of proteins significantly modulated during whole blood storage. STRING analysis of the proteins significantly modulated during whole blood storage revealed a functionally interconnected network enriched in immune- and defense-related biological processes. Nodes highlighted in blue identify proteins associated with the Gene Ontology term regulation of immune response, whereas nodes highlighted in red identify proteins associated with defense response.

Gene Ontology enrichment analysis identified a significant overrepresentation of biological processes related to regulation of immune response (FDR = 6.02 × 10^−7^) and defense response (FDR = 2.01× 10^−8^) (Figure 3). Together, these data indicate that storage-associated changes in the plasma proteome mainly involve coordinated pathways linked to immune and host defense functions.

### 3.4. Whole Blood Storage Affects Red Blood Cells Hemorheological Properties

**Figure 4:**
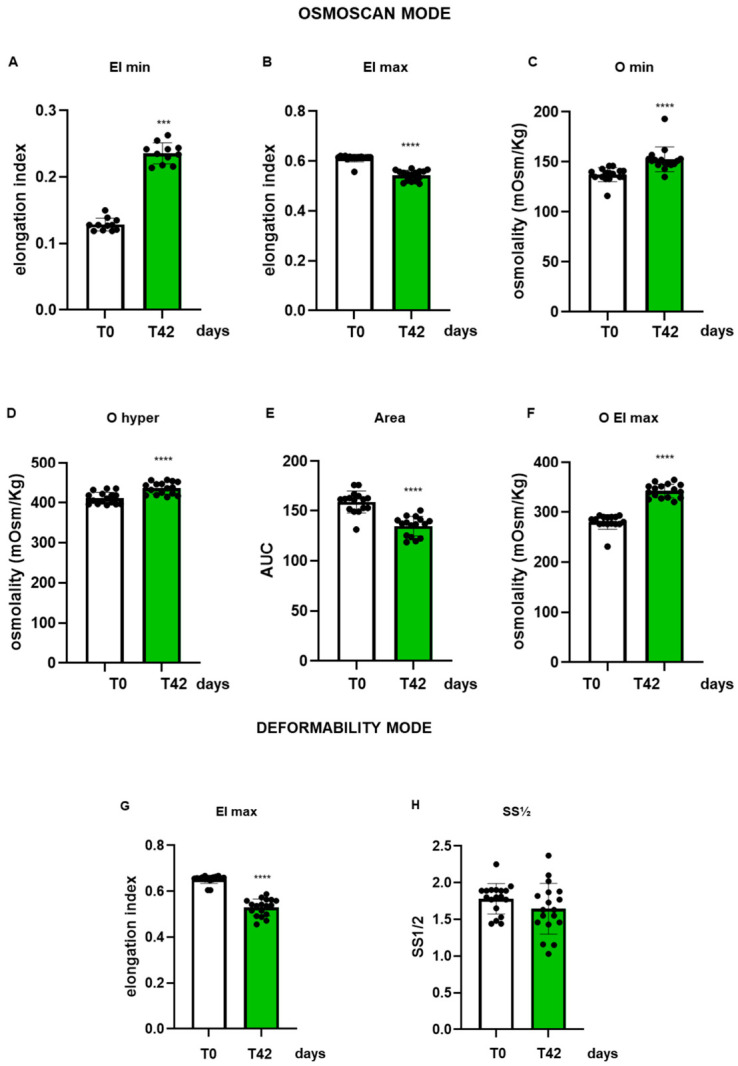
Changes in RBC osmoscan and deformability parameters after whole blood storage. Osmoscan parameters were measured in RBCs at baseline (T0, white) and after 42 days of whole blood storage at 4 °C (T42, green) using the LORRCA system. (**A**) Elongation index minimum (EI min) n = 11. (**B**) Elongation index maximum (EI max) n = 16. (**C**) Minimum osmolality (O min) n = 16. (**D**) Hypertonic osmolality (O hyper) n = 16. (**E**) Area under the curve (AUC) n = 16. (**F**) Osmolality at maximal elongation index (O EI max). n = 16. RBC deformability parameters were measured using the deformability mode of the LORRCA system in whole blood samples collected at baseline (T0, white) and after 42 days of storage at 4 °C (T42, green). (**G**) Elongation index maximum (EI max) measured with increasing shear stress n = 18. (**H**) Shear stress required to achieve half-maximal deformation (SS½). n = 18. *** *p* < 0.001; **** *p* < 0.0001.

Whole blood storage significantly altered RBC hemorheological properties, including deformability and cell stability. Six parameters were evaluated by osmoscan analysis using the state-of-the-art LORRCA system (Figure 4). After 42 days of refrigerated storage, RBC deformability was profoundly modified: cells were more deformable at low osmolalities, as indicated by the increase in EI min, but less deformable at isotonic osmolality, as shown by the reduction in EI max (Figure 4A,B). In addition, both O min and O hyper were significantly increased (Figure 4C,D), indicating greater osmotic fragility and a tendency toward RBC overhydration. These changes were accompanied by a significant reduction in the area under the osmoscan) curve (Figure 4E). Moreover, after 42 days of storage, maximal RBC deformability was reached at higher osmolality than at baseline, as reflected by the increase in O EI max (Figure 4F). Overall, these findings indicate that prolonged whole blood storage impairs RBC osmotic behavior and deformability. 

RBC deformability was also assessed using the deformability mode of the LORRCA system. An increasing shear stress (0.3–30 Pa) was applied to the whole blood samples at T0 and T42 and the EI max and SS½ were recorded (Figure 4G,H). After storage, EI max was significantly reduced compared with T0, indicating impaired RBC deformability (Figure 4G). By contrast, no significant difference was observed in SS½ between the two time points (Figure 4H). Overall, these results indicate that prolonged whole blood storage reduces RBC deformability without significantly affecting the shear stress required to achieve half-maximal elongation. 

### 3.5. N-Acetylcysteine Regenerates Mercaptoalbumin and Reduces Thiolated Albumin

**Figure 5:**
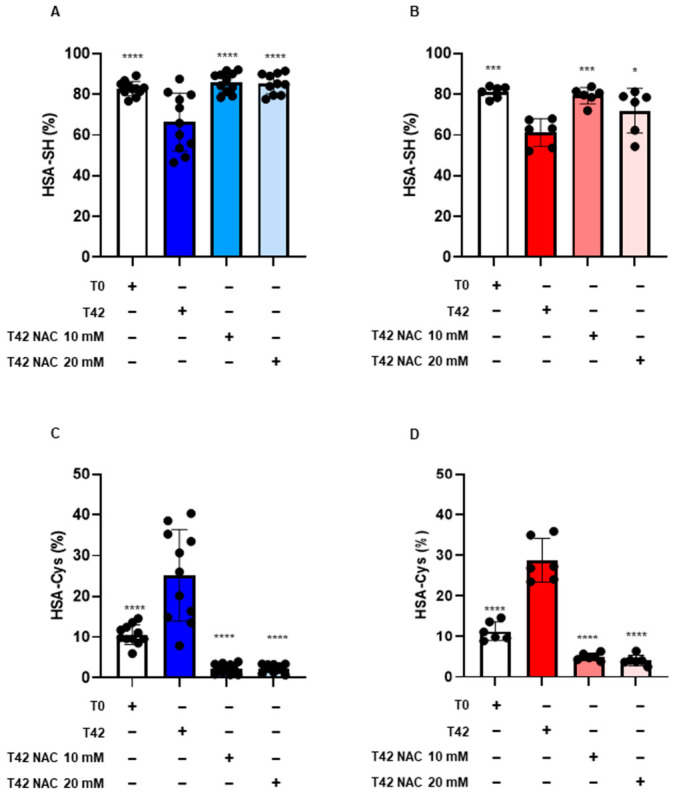
NAC preserves albumin redox status during whole blood storage. HSA proteoforms were measured at baseline (T0, white) and after 42 days (dark blue or dark red) of whole blood storage at 4 °C in the absence or presence of NAC 10 mM (blue or red) or 20 mM (light blue or light red). (**A**,**B**) Plasma levels of mercaptoalbumin (HSA-SH) at T0 and after 42 days of whole blood storage (T42) in the absence or presence of NAC. In (**A**), NAC was added at T0 and every 10 days thereafter (n = 11), whereas in (**B**) NAC was added only once at T0 (n = 6). (**C**,**D**) Plasma levels of thiolated albumin (HSA-Cys) at T0 and T42 in the absence or presence of NAC. In (**C**), NAC was added at T0 and every 10 days thereafter (n = 11), whereas in (**D**), NAC was added only once at T0 (n = 6). * *p* < 0.05; *** *p* < 0.001; **** *p* < 0.0001 vs. T42.

The addition of NAC at T0 and subsequently every 10 days during storage, at either 10 mM or 20 mM, restored HSA-SH and markedly reduced the levels of HSA-Cys (Figure 5A,C). No significant differences were observed between 10 mM and 20 mM NAC, suggesting that 10 mM is sufficient to achieve the maximal effect. A comparable pattern was also observed when NAC was added only once at T0 (Figure 5B,D). Taken together, these findings indicate that NAC effectively preserves plasma HSA redox balance during whole blood storage.

### 3.6. N-Acetylcysteine Reduces Hemoglobin Oxidation During Whole Blood Storage

**Figure 6:**
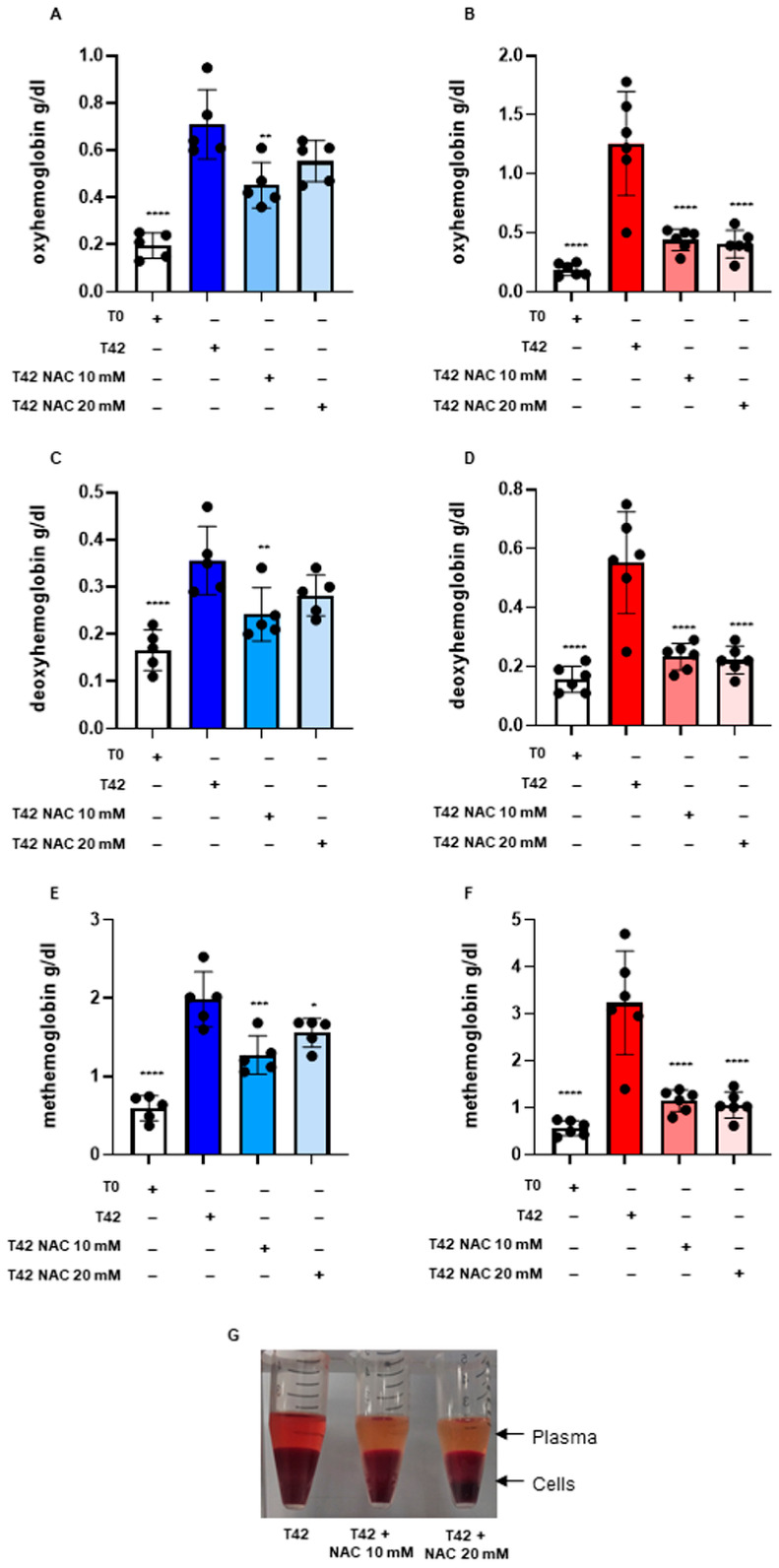
Plasma levels of oxyhemoglobin, deoxyhemoglobin, and methemoglobin were measured by a NanoDrop spectrophotometer at T0 (white) and after 42 days (dark blue or dark red) of whole blood storage at 4 °C in the absence or presence of NAC 10 mM (blue or red) or 20 mM (light blue or light red). (**A**,**B**) Levels of oxyhemoglobin at T0 and T42 in the absence or presence of NAC added (**A**) at T0 and every 10 days (n = 5), (**B**) only once at T0 (n = 6). (**C**,**D**) Levels of deoxyhemoglobin at T0 and T42 in the absence or presence of NAC added (**C**) at T0 and every 10 days (n = 5), (**D**) only once at T0 (n = 6). (**E**,**F**) levels of methemoglobin at T0 and T42 in the absence and in the presence of NAC added (**E**) at T0 and every 10 days (n = 5), (**F**) only once at T0 (n = 6). (**G**) Representative image of hemolysis at T42 in the absence or in the presence of NAC. * *p* < 0.05; ** *p* < 0.01; *** *p* < 0.001; **** *p* < 0.0001 vs. T42.

The addition of NAC to whole blood significantly attenuated the hemolysis of RBCs stored for 42 days. In particular, levels of oxyhemoglobin (Figure 6A,B), deoxyhemoglobin (Figure 6C,D) and methemoglobin (Figure 6E,F) were all markedly lower in NAC-treated samples than in the absence of NAC. This effect was not dose-dependent, as the maximal reduction was already observed at 10 mM NAC. Notably, NAC administration only once at T0 (Figure 6B,D,F) appeared to be more effective than repeated supplementation every 10 days (Figure 6A,C,E). Thus, these findings indicate that NAC limits hemoglobin oxidation and reduces the accumulation of free hemoglobin species during prolonged whole blood storage.

The marked increase in plasma levels of oxyhemoglobin and its derivatives, deoxyhemoglobin and methemoglobin, after storage, suggests damage to the RBC membrane with a subsequent hemolysis. The presence of NAC exogenously added to whole blood markedly reduces the hemolysis (−32.7% ± 19.8 and 16.5% ±23.6 for NAC 10 and 20 mM added at T0 and every 10 days, respectively; −61.7% ± 11.0 and 65.6% ± 6.3 for NAC 10 and 20 mM only once at T0. Figure 6G reports a representative image.

### 3.7. N-Acetylcysteine Partially Preserves the Plasma Proteomic Profile During Whole Blood Storage

**Figure 7:**
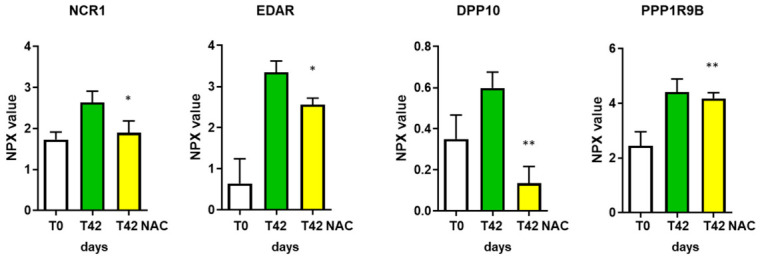
Effect of NAC on plasma proteomic profile. Proteomic profile was detected by PEA technology at T0 (white) and after 42 days of refrigerated storage in the absence (green) or in the presence of NAC 10 mM (yellow). n = 3. * *p* < 0.05; ** *p* < 0.01 vs. T42.

The addition of 10 mM NAC to whole blood partially preserved the plasma proteomic profile during storage. NAC modulated several proteins that were altered after whole blood storage. Statistical significance was reached only for a limited number of proteins, namely natural cytotoxicity triggering receptor 1 (NCR1), ectodysplasin-A receptor (EDAR), Dipeptidyl peptidase-like 10 (DPP10), and protein phosphatase 1 regulatory subunit 9B (PPP1R9B) (Figure 7), likely because of the small sample size (n = 3) and inter-individual variability. However, 11 additional proteins showed a trend toward the baseline levels measured at T0 (Figure S1, highlighted in red). To further characterize the proteins modulated by NAC, STRING network analysis was performed using a first-shell setting with up to 10 additional interactors. The analysis suggested enrichment for terms related to T-cell activation (FDR = 0.0317) and cell adhesion (FDR = 0.0086) (Figure S2). Overall, these data indicate that NAC partially counteracts storage-associated changes in the plasma proteomic profile.

### 3.8. N-Acetylcysteine Partially Preserves Red Blood Cell Hemorheological Properties During Whole Blood Storage

**Figure 8:**
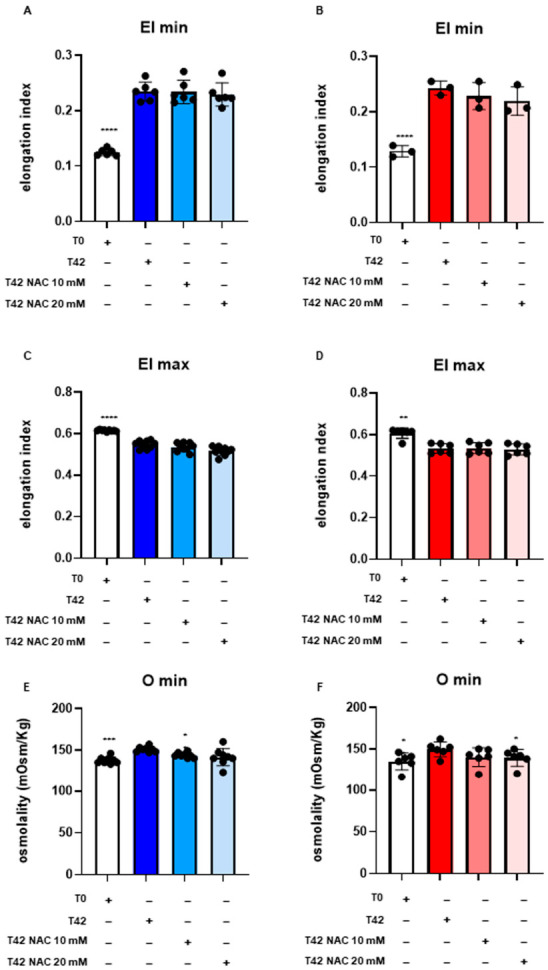

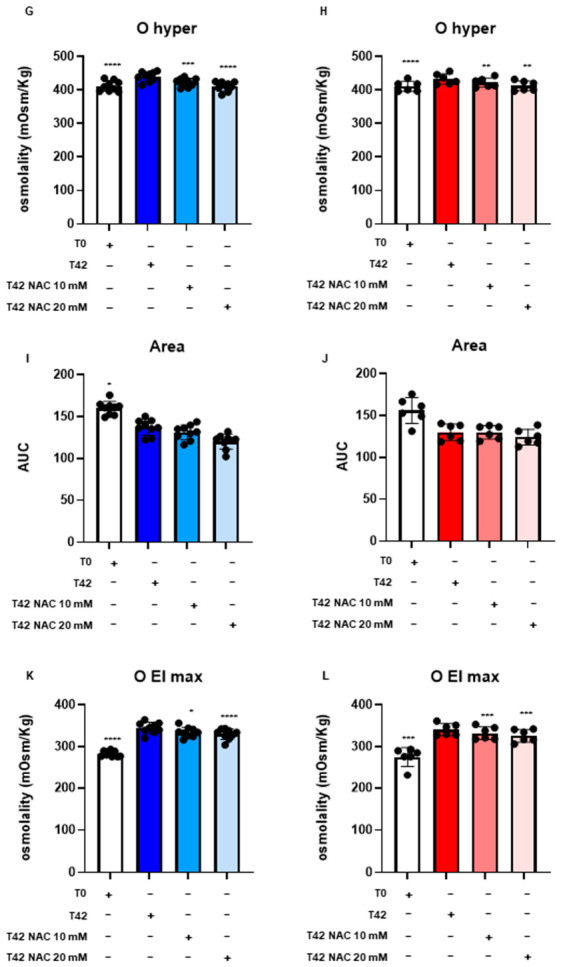
Effects of NAC on RBC osmoscan parameters during whole blood storage. Osmoscan parameters were measured in RBCs at baseline (T0, white) and after 42 days of whole blood storage at 4 °C (T42, dark blue or dark red) in the absence or presence of NAC 10 mM (blue or red) or 20 mM (light blue or light red). (**A**,**B**) Elongation index minimum (EI min) with NAC added (**A**) at T0 and every 10 days (n = 6), or (**B**) only once at T0 (n = 3). (**C**,**D**) Elongation index maximum (EI max) with NAC added (**C**) at T0 and every 10 days (n = 9), or (**D**) only once at T0 (n = 6). (**E**,**F**) Minimum osmolality (O min) with NAC added (**E**) at T0 and every 10 days (n = 8), or (**F**) only once at T0 (n = 6). (**G**,**H**) Hypertonic osmolality (O hyper) with NAC added (**G**) at T0 and every 10 days (n = 9), or (**H**) only once at T0 (n = 6). (**I**,**J**) Area under the curve (AUC) with NAC added (**I**) at T0 and every 10 days (n = 9), or (**J**) only once at T0 (n = 6). (**K**,**L**) Osmolality at maximal elongation index (O EI max) with NAC added (**K**) at T0 and every 10 days (n = 9), or (**L**) only once at T0 (n = 6). * *p* < 0.05; ** *p* < 0.01; *** *p* < 0.001; **** *p* < 0.0001 vs. T42.

NAC supplementation partially preserved RBC hemorheological properties during 42 days of storage (Figure 8). While NAC had no significant effect on EI min and EI max (Figure 8A–D), its protective effect was particularly evident for osmoscan parameters related to osmotic fragility and cell hydration, including O min, O hyper, and O EI max (Figure 8E–H,K,L), both with NAC added at T0 and subsequently every 10 days during storage (Figure 8E,G,K), and with NAC added only once at T0 (Figure 8F,H,L). Similar results were observed with both 10 mM and 20 mM NAC, indicating no clear dose-dependent effect under these experimental conditions. Taken together, these findings suggest that NAC partially attenuates storage-associated alterations in RBC osmotic behavior.

By contrast, NAC supplementation, at either 10 mM or 20 mM, and regardless of whether it was administered once or repeatedly, did not significantly affect the EI max parameter measured by the LORRCA deformability mode. This finding indicates that NAC had a greater impact on osmotic fragility-related parameters than on shear stress-dependent RBC deformability.

## 4. Discussion

Whole blood storage is associated with biochemical and biomechanical alterations, collectively referred to as storage lesions, which may compromise cell viability and function. Several factors may contribute to these changes; however, oxidative stress is considered a major determinant. Accordingly, several strategies have been explored to counteract oxidative damage or to strengthen intracellular or extracellular antioxidant defenses. Supplementation with antioxidant molecules, such as ascorbic acid, vitamin E, and NAC, either alone or in combination, has been proposed as a means of improving RBC storability [28,32,38,39].

The present study shows that 42 days of refrigerated whole blood storage are associated with coordinated oxidative and functional alterations involving both the plasma and RBC compartments. In particular, storage was associated with a shift in HSA redox state toward the oxidized proteoform HSA-Cys, increased hemoglobin oxidation and spontaneous hemolysis, changes in the plasma proteomic profile, and impairment of RBC rheological properties. Within this framework, NAC attenuated several of these alterations, suggesting that antioxidant supplementation may partially preserve whole blood quality during storage.

One of the main findings of this study is that oxidative stress during whole blood storage is detectable not only at the cellular level but also in the plasma compartment. The redox state of circulating HSA is considered a marker of systemic oxidative stress [40], and here, we show that after 42 days of whole blood storage, plasma levels of the native reduced proteoform HSA are markedly decreased, with a concomitant increase in the oxidized proteoform HSA-Cys. This result is particularly relevant because it indicates that storage-related oxidative injury progressively affects the extracellular redox environment of whole blood. In a previous paper, we demonstrated that NAC enhances plasma antioxidant activity by restoring HSA Cys34 through a selective thiol-disulfide mechanism [41]. That effect was rapid, becoming evident after a 60 min incubation at 37 °C. In the present study, we extended this observation by showing that the antioxidant action of NAC is also evident at 4 °C and is maintained throughout prolonged storage. The comparable effect observed after a single supplementation at T0 and after repeated additions every 10 days further suggests that NAC exerts a persistent protective effect on the plasma redox milieu.

The erythrocyte findings are consistent with the view that hemoglobin oxidation is a key event in the development of storage-related RBC damage [42]. During storage, the progressive decline in antioxidant capacity likely favors hemoglobin oxidation, which in turn contributes to membrane injury, protein aggregation, microvesicle release, and ultimately hemolysis [24,43]. Indeed, experiments performed on hemoglobin-free erythrocyte ghosts exposed to H_2_O_2_ have suggested that the first target of oxidative damage is the intracellular content, particularly hemoglobin, which interacts with membrane phospholipids and proteins, including spectrin and band 3, promotes the release of hemoglobin-containing membrane microvesicles, and ultimately leads to hemolysis [42,44,45]. In addition, hemoglobin is both a target of oxidation and an important source of superoxide anion generation, thereby propagating oxidative injury within RBCs [46]. Taken together, these observations support the view that hemoglobin oxidation is an upstream event in the development of storage-related RBC damage. Several antioxidants with different mechanisms of action have been proposed to protect RBCs from oxidative injury during storage, including NAC alone or in combination. In particular, supplementation with NAC and vitamin C has been shown to counteract oxidative stress-related lesions at different levels, including glutathione replenishment, modulation of the glycolytic pathway, and reduction in hemolysis [32]. In this context, our results indicate that NAC significantly limits hemoglobin oxidation and reduces spontaneous hemolysis, in line with previous evidence on its protective effect against oxidative RBC damage. Overall, these findings support the interpretation that NAC attenuates, at least in part, the oxidative cascade linking hemoglobin oxidation to membrane destabilization and hemolysis.

Whole blood storage also affected the plasma proteomic profile, consistent with previous evidence showing that storage is accompanied by progressive remodeling of the extracellular milieu. Within this context, NAC supplementation modified part of this storage-associated proteomic pattern. Among the significantly modulated proteins, NCR1 and PPP1R9B are implicated in pathways related to immune-cell activation and cell–cell interaction, whereas the relevance of EDAR, a membrane receptor involved in the reprogramming of stem cells, and DPP10, a catalytically inactive member of the dipeptidyl peptidase family, in the context of whole blood storage remains less clear. Therefore, these findings should be considered exploratory and not overinterpreted at the mechanistic level. Briefly, NAC supplementation during storage also affected the levels of 11 additional proteins that tended to return toward baseline values. STRING network analysis further indicated that these proteins are related to T-cell activation and cell adhesion. Given the limited number of significantly modulated proteins, these findings should be interpreted with caution; however, they suggest that NAC may influence not only redox balance and hemolysis, but also part of the storage-associated remodeling of the plasma milieu.

Storage also significantly impaired RBC rheological behavior, as shown by the state-of-the-art LORRCA-derived parameters. In our study, the main effects of NAC were observed in osmoscan-related variables, particularly O min, O hyper, and O EI max, whereas the recovery was only partial. Rather than indicating a complete prevention of storage-related injury, this pattern suggests that NAC mainly preserves aspects of membrane homeostasis that are reflected by osmotic fragility and cell hydration. The attenuation of the increase in O min is consistent with reduced susceptibility to spontaneous hemolysis during storage, whereas the prevention of O hyper increase supports a partial preservation of RBC hydration state and membrane integrity [47]. Thus, the rheological data are coherent with the biochemical findings and indicate that NAC mitigates, but does not abolish, the functional deterioration associated with prolonged refrigeration.

Some limitations of the present study should be acknowledged. First, this is an ex vivo study performed under experimental storage conditions, and the findings therefore cannot be directly extrapolated to clinical transfusion settings. Second, some analyses, particularly the proteomic assessment, were performed on a limited number of samples, which reduces statistical power and warrants cautious interpretation. Third, although NAC attenuated several storage-associated alterations, the study was not designed to determine whether these effects translate into improved post-transfusion recovery or function in vivo. Moreover, no consistent advantage of 20 mM over 10 mM NAC was observed across the investigated parameters, suggesting that the antioxidant effect may approach saturation at the lower concentration under our experimental conditions. Since concentrations below 10 mM were not evaluated, the present study cannot identify the minimum effective dose. Future dose–response studies including lower NAC concentrations will be important to define the lowest concentration capable of preserving whole blood quality while facilitating the clinical translation of this strategy. Despite these limitations, the integrated evaluation of HSA redox state, hemoglobin oxidation, plasma proteomic remodeling, and RBC rheology provides a coherent picture of oxidative injury during whole blood storage and identifies NAC as a potentially useful strategy to limit this process.

## 5. Conclusions

This study provides an integrated evaluation of the major changes occurring after 42 days of refrigerated whole blood storage. These changes include a reduction in the antioxidant proteoform HSA-SH, a concomitant increase in the oxidized proteoform HSA-Cys, increased spontaneous hemolysis, alterations in the plasma proteomic profile, and impaired rheological properties of RBCs. Together, these findings indicate that prolonged storage affects both the plasma compartment and RBC integrity and function. We further show that the addition of exogenous NAC before storage attenuates several of these alterations by preserving the plasma redox environment, limiting hemoglobin oxidation and hemolysis, and partially maintaining RBC rheological behavior. Although additional studies are required to define the optimal NAC concentration and to validate these findings in clinically relevant transfusion settings, the present results provide a rationale for further investigation of antioxidant supplementation as a strategy to mitigate storage-associated alterations during whole blood storage.

## Data Availability

Data collected in the study will be made available using the data repository Zenodo (https://zenodo.org) with restricted access upon request to direzione.scientifica@ccfm.it. Any remaining information can be obtained from the corresponding author upon reasonable request.

## Supplementary Materials

The following supporting information can be downloaded at: https://www.mdpi.com/article/10.3390/antiox15070858/s1, Figure S1: Effect of n-acetylcysteine on proteins significantly altered after whole blood storage; Figure S2: STRING network analysis of plasma proteins modulated by NAC during whole blood storage; Table S1: Target proteins of immune response panel; Table S2: Plasma proteomic profile at baseline and after whole blood storage.
