# Supplementary material for: N-Acetylcysteine Attenuates Oxidative Stress and Preserves Red Blood Cell Quality During Whole Blood Storage
Source: Antioxidants (Basel). 2026 Jul 8;15(7):858. doi: 10.3390/antiox15070858 (PMC13404811; doi:10.3390/antiox15070858)
Supplement: Supplementary file 1 [file antioxidants-15-00858-s001.zip › Table S1.pdf]

Table S1: Target proteins of immune response panel

Uniprot ID	Gene name	Biomarker
P28845	HSD11B1	Corticosteroid 11-beta-dehydrogenase isozyme 1
P30044	PRDX5	Peroxiredoxin-5, mitochondrial
P30048	PRDX3	Thioredoxin-dependent peroxide reductase, mitochondrial
P34130	NTF4	Neurotrophin-4
P35240	NF2	Merlin
P42701	IL12RB1	Interleukin-12 receptor subunit beta-1
P48061	CXCL12	Stromal cell-derived factor 1
P48740	MASP1	Mannan-binding lectin serine protease 1
P50135	HNMT	Histamine N-methyltransferase
P51617	IRAK1	Interleukin-1 receptor-associated kinase 1
P51671	CCL11	Eotaxin
P52823	STC1	Stanniocalcin-1
P58499	FAM3B	Protein FAM3B
P63241	EIF5A	Eukaryotic translation initiation factor 5A-1
P78310	CXADR	Coxsackievirus and adenovirus receptor
P78362	SRPK2	SRSF protein kinase 2
P78410	BTN3A2	Butyrophilin subfamily 3 member A2
Q01151	CD83	CD83 antigen
Q03431	PTH1R	Parathyroid hormone/parathyroid hormone-related peptide receptor
Q04637	EIF4G1	Eukaryotic translation initiation factor 4 gamma 1
Q04759	PRKCQ	Protein kinase C theta type
Q05084	ICA1	Islet cell autoantigen 1
P23229	ITGA6	Integrin alpha-6
P27540	ARNT	Aryl hydrocarbon receptor nuclear translocator
P19474	TRIM21	E3 ubiquitin-protein ligase TRIM21
O14867	BACH1	Transcription regulator protein BACH1
O43597	SPRY2	Protein sprouty homolog 2
O43736	ITM2A	Integral membrane protein 2A
O60449	LY75	Lymphocyte antigen 75
O60880	SH2D1A	SH2 domain-containing protein 1A

O75475	PSIP1	PC4 and SFRS1-interacting protein
O76036	NCR1	Natural cytotoxicity triggering receptor 1
O94992	HEXIM1	Protein HEXIM1
O95786	RIGI	Antiviral innate immune response receptor RIG-I
P05113	IL5	Interleukin-5
P22301	IL10	Interleukin-10
O00273	DFFA	DNA fragmentation factor subunit alpha
P05412	JUN	Transcription factor AP-1
P08727	KRT19	Keratin, type I cytoskeletal 19
P09038	FGF2	Fibroblast growth factor 2
P10747	CD28	T-cell-specific surface glycoprotein CD28
P14317	HCLS1	Hematopoietic lineage cell-specific protein
P15514	AREG	Amphiregulin
P16278	GLB1	Beta-galactosidase
P16455	MGMT	Methylated-DNA--protein-cysteine methyltransferase
P18564	ITGB6	Integrin beta-6
P18627	LAG3	Lymphocyte activation gene 3 protein
P05231	IL6	Interleukin-6
P52294	KPNA1	Importin subunit alpha-5
Q00978	IRF9	Interferon regulatory factor 9
Q8N608	DPP10	Inactive dipeptidyl peptidase 10
Q8IU57	IFNLR1	Interferon lambda receptor 1
Q7Z6M3	MILR1	Allergin-1
Q6ZUJ8	PIK3AP1	Phosphoinositide 3-kinase adapter protein 1
Q6UXB4	CLEC4G	C-type lectin domain family 4 member G
Q6EIG7	CLEC6A	C-type lectin domain family 6 member A
Q6DN72	FCRL6	Fc receptor-like protein 6
Q15661	TPSAB1	Tryptase alpha/beta-1
Q8NHJ6	LILRB4	Leukocyte immunoglobulin-like receptor subfamily B member 4
Q15517	CDSN	Corneodesmosin
Q14203	DCTN1	Dynactin subunit 1
Q13574	DGKZ	Diacylglycerol kinase zeta

Q13490	BIRC2	Baculoviral IAP repeat-containing protein 2
Q13241	KLRD1	Natural killer cells antigen CD94
Q12968	NFATC3	Nuclear factor of activated T-cells, cytoplasmic 3
Q12933	TRAF2	TNF receptor-associated factor 2
Q07065	CKAP4	Cytoskeleton-associated protein 4
Q06830	PRDX1	Peroxiredoxin-1
Q14435	GALNT3	Polypeptide N-acetylgalactosaminyltransferase 3
Q05516	ZBTB16	Zinc finger and BTB domain-containing protein 16
Q8WTT0	CLEC4C	C-type lectin domain family 4 member C
Q92844	TANK	TRAF family member-associated NF-kappa-B activator
Q9Y2J8	PADI2	Protein-arginine deiminase type-2
Q9UQV4	LAMP3	Lysosome-associated membrane glycoprotein 3
Q9UQQ2	SH2B3	SH2B adapter protein 3
Q9UNE0	EDAR	Tumor necrosis factor receptor superfamily member EDAR
Q9UN19	DAPP1	Dual adapter for phosphotyrosine and 3-phosphotyrosine and 3-phosphoinositide
Q9UMR7	CLEC4A	C-type lectin domain family 4 member A
Q9UKX5	ITGA11	Integrin alpha-11
Q9UHC6	CNTNAP2	Contactin-associated protein-like 2
Q8WXI8	CLEC4D	C-type lectin domain family 4 member D
Q9NWZ3	IRAK4	Interleukin-1 receptor-associated kinase 4
Q9HCM2	PLXNA4	Plexin-A4
Q9GZT9	EGLN1	Egl nine homolog 1
Q9C035	TRIM5	Tripartite motif-containing protein 5
Q9BXN2	CLEC7A	C-type lectin domain family 7 member A
Q96SB3	PPP1R9B	Neurabin-2
Q96PD2	DCBLD2	Discoidin, CUB and LCCL domain-containing protein 2
Q96P31	FCRL3	Fc receptor-like protein 3
Q96DB9	FXYD5	FXYD domain-containing ion transport regulator 5
Q9NP99	TREM1	Triggering receptor expressed on myeloid cells 1
Q9Y3P8	SIT1	Signaling threshold-regulating transmembrane adapter 1
