# Supplementary material for: N-Acetylcysteine Attenuates Oxidative Stress and Preserves Red Blood Cell Quality During Whole Blood Storage
Source: Antioxidants (Basel). 2026 Jul 8;15(7):858. doi: 10.3390/antiox15070858 (PMC13404811; doi:10.3390/antiox15070858)
Supplement: Supplementary file 1 [file antioxidants-15-00858-s001.zip › Table S2.pdf]

Table S2: Plasma proteomic profile at baseline and after whole blood storage

**Protein name**	**Uniprot ID**	**Gene name**	**NPX value T0**	**NPX value T42**	**p-value**
**Tumor necrosis factor receptor superfamily member EDAR**	**Q9UNE0**	EDAR	0.6 [0.4; 0.9]	3.2 [3.1; 3.7]	**0.0001**
**Peroxiredoxin-5**	**P30044**	PRDX5	3.1 [3.0; 3.4]	7.7 [7.4; 8.0]	**0.0017**
**Protein sprouty homolog 2**	**O43597**	SPRY2	-0.0 [-0.5; 0.8]	1.8 [1.3; 2.5]	**0.0017**
**Integral membrane protein 2A**	**O43736**	ITM2A	2.3 [2.2; 2.7]	1.7 [1.5; 2.1]	**0.0020**
**Signaling threshold-regulating transmembrane adapter 1**	**Q9Y3P8**	SIT1	2.5 [2.1; 2.9]	5.7 [5.4; 6.2]	**0.0031**
**Thioredoxin-dependent peroxide reductase**	**P30048**	PRDX3	-0.7 [-0.9; -0.6]	2.2 [2.0; 2.7]	**0.0032**
**C-type lectin domain family 4 member A**	**Q9UMR7**	CLEC4A	3.0 [2.6; 3.2]	4.6 [4.4; 5.2]	**0.0035**
**Eotaxin**	**P51671**	CCL11	7.6 [7.2; 7.7]	9.0 [8.7; 9.4]	**0.0036**
**Integrin alpha-6**	**P23229**	ITGA6	-0.0 [-0.5; 0.02]	5.1 [4.6; 5.9]	**0.0058**
**Mannan-binding lectin serine protease 1**	**P48740**	MASP1	-0.3 [0.4; -0.2]	1.4 [1.1; 1.6]	**0.0062**
**Plexin-A4**	**Q9HCM2**	PLXNA4	4.2 [3.6; 5.0]	7.7 [7.4; 8.2]	**0.0067**
**SH2 domain-containing protein 1A**	**O60880**	SH2D1A	0.4 [0.2; 1.2]	6.2 [5.9; 7.0]	**0.0068**
**Coxsackievirus and adenovirus receptor**	**P78310**	CXADR	0.7 [0.5; 0.8]	1.2 [1.0; 1.5]	**0.0080**
**Stromal cell-derived factor 1**	**P48061**	CXCL12	0.5 [-0.01; 0.6]	0.9 [0.3; 0.9]	**0.0083**
**Triggering receptor expressed on myeloid cells 1**	**Q9NP99**	TREM1	1.2 [-0.1; 1.3]	3.0 [2.1; 3.8]	**0.0096**
**Stanniocalcin-1**	**P52823**	STC1	4.8 [4.4; 5.2]	4.0 [3.7; 4.7]	**0.0101**
**Antiviral innate immune response receptor RIG-I**	**O95786**	DDX58	1.1 [1.0; 2.0]	7.8 [7.0; 9.2]	**0.0123**
**Natural cytotoxicity triggering receptor 1**	**O76036**	NCR1	1.7 [1.5; 1.9]	2.7 [2.3; 2.9]	**0.0125**
**SRSF protein kinase 2**	**P78362**	SRPK2	-0.6 [-0.7; 0.9]	4.4 [3.7; 4.6]	**0.0126**
**Egl nine homolog 1**	**Q9GZT9**	EGLN1	-0.0 [-0.4; 0.6]	5.3 [3.8; 6.2]	**0.0140**
**DNA fragmentation factor subunit alpha**	**O00273**	DFFA	2.9 [2.6; 3.3]	6.0 [6.0; 6.6]	**0.0141**
**Inactive dipeptidyl peptidase 10**	**Q8N608**	DPP10	0.4 [0.2; 0.5]	0.6 [0.5; 0.6]	**0.0147**
**Interleukin-1 receptor-associated kinase 4**	**Q9NWZ3**	IRAK4	0.6 [0.5; 0.6]	3.8 [3.1; 4.4]	**0.0164**
**Islet cell autoantigen 1**	**Q05084**	ICA1	-0.5 [-0.6; -0.1]	2.3 [1.4; 2.4]	**0.0168**
**Leukocyte immunoglobulin-like receptor subfamily B member 4**	**Q8NHJ6**	LILRB4	1.3 [0.8; 1.3]	0.7 [0.4; 0.9]	**0.0169**
**Fibroblast growth factor 2**	**P09038**	FGF2	0.1 [-0.7; 0.3]	2.3 [0.8; 2.5]	**0.0173**
**Protein HEXIM1**	**O94992**	HEXIM1	3.0 [2.7; 3.8]	7.1 [6.7; 7.8]	**0.0180**
**Eukaryotic translation initiation factor 4 gamma 1**	**Q04637**	EIF4G1	3.2 [3.1; 4.4]	7.6 [7.2; 8.3]	**0.0196**
**Interferon regulatory factor 9**	**Q00978**	IRF9	0.7 [0.7; 1.1]	5.4 [4.8; 7.0]	**0.0198**
**PC4 and SFRS1-interacting protein**	**O75475**	PSIP1	1.7 [0.7; 2.8]	7.2 [6.8; 7.2]	**0.0203**
**Baculoviral IAP repeat-containing protein 2**	**Q13490**	BIRC2	-0.5 [-0.6; -0.0]	1.2 [0.9; 1.5]	**0.0210**
**Hematopoietic lineage cell-specific protein**	**P14317**	HCLS1	2.5 [1.8; 3.7]	6.6 [6.5; 6.6]	**0.0213**
**Integrin alpha-11**	**Q9UKX5**	ITGA11	0.5 [0.3; 1.2]	2.2 [1.7; 2.6]	**0.0232**
**Methylated-DNA--protein-cysteine methyltransferase**	**P16455**	MGMT	3.9 [3.1; 4.8]	7.6 [7.5; 7.8]	**0.0238**
**Neurabin-2**	**Q96SB3**	PPP1R9B	2.4 [1.9; 3.0]	4.5 [4.2; 4.5]	**0.0251**
**Transcription regulator protein BACH1**	**O14867**	BACH1	0.8 [0.7; 1.8]	4.0 [3.1; 4.5]	**0.0254**
**Diacylglycerol kinase zeta**	**Q13574**	DGKZ	-1.1 [-1.3; -0.8]	0.9 [0.3; 1.5]	**0.0262**
**Merlin**	**P35240**	NF2	-2.0 [-2.8; -1.7]	1.4 [1.0; 1.9]	**0.0280**
**Interleukin-1 receptor-associated kinase 1**	**P51617**	IRAK1	0.7 [0.7; 1.3]	2.7 [2.4; 3.2]	**0.0291**
**Polypeptide N-acetylgalactosaminyltransferase 3**	**Q14435**	GALNT3	1.4 [1.0; 1.4]	1.9 [1.3; 2.0]	**0.0304**

**Phosphoinositide 3-kinase adapter protein 1**	**Q6ZUJ8**	PIK3AP1	2.8 [2.0; 2.9]	5.8 [5.8; 6.9]	**0.0306**
**Lymphocyte antigen 75**	**O60449**	LY75	1.3 [1.1; 1.8]	1.7 [1.7; 2.3]	**0.0310**
**Dual adapter for phosphotyrosine and 3-phosphotyrosine and 3-phosphoinositide**	**Q9UN19**	DAPP1	1.1 [-0.3; 1.4]	3.0 [2.9; 3.5]	**0.0330**
**Histamine N-methyltransferase**	**P50135**	HNMT	7.4 [6.7; 7.5]	9.9 [8.8; 11]	**0.0339**
**E3 ubiquitin-protein ligase TRIM21**	**P19474**	TRIM21	0.8 [0.2; 1.0]	4.5 [3.9; 5.9]	**0.0345**
**Nuclear factor of activated T-cells, cytoplasmic 3**	**Q12968**	NFATC3	-0.8 [-0.9; -0.3]	0.8 [0.5; 1.3]	**0.0348**
**Tripartite motif-containing protein 5**	**Q9C035**	TRIM5	1.3 [0.8; 1.9]	3.9 [3.4; 4.3]	**0.0362**
**SH2B adapter protein 3**	**Q9UQQ2**	SH2B3	2.5 [1.8; 3.1]	4.0 [3.7; 4.3]	**0.0379**
**Peroxiredoxin-1**	**Q06830**	PRDX1	2.0 [1.5; 3.6]	6.2 [5.2; 6.9]	**0.0480**
**T-cell-specific surface glycoprotein CD28**	**P10747**	CD28	0.4 [0.2; 0.9]	1.6 [1.1; 1.8]	**0.0482**
**C-type lectin domain family 7 member A**	**Q9BXN2**	CLEC7A	2.4 [1.5; 2.5]	3.4 [2.2; 4.0]	**0.0488**
**Butyrophilin subfamily 3 member A2**	**P78410**	BTN3A2	1.4 [1.3; 1.8]	2.5 [2.5; 3.0]	**0.0497**
**Interleukin-5**	**P05113**	IL5	-0.6 [-1.0; 1.2]	-0.4 [-0.8; 1.4]	0.0502
**TRAF family member-associated NF-kappa-B activator**	**Q92844**	TANK	1.4 [0.5; 1.5]	3.0 [2.5; 3.1]	0.0545
**Interleukin-12 receptor subunit beta-1**	**P42701**	IL12RB1	0.9 [0.7; 1.2]	1.2 [1.2; 1.5]	0.0603
**Dynactin subunit 1**	**Q14203**	DCTN1	3.4 [2.8; 3.5]	6.7 [5.2; 7.5]	0.0642
**TNF receptor-associated factor 2**	**Q12933**	TRAF2	2.6 [1.8; 2.8]	4.5 [4.5; 5.6]	0.0658
**Zinc finger and BTB domain-containing protein 16**	**Q05516**	ZBTB16	0.4 [-0.6; 1.4]	2.8 [2.3; 2.9]	0.0678
**Parathyroid hormone/parathyroid hormone-related peptide receptor**	**Q03431**	PTH1R	2.5 [2.4; 2.6]	1.9 [1.9; 2.3]	0.0711
**Protein kinase C theta type**	**Q04759**	PRKCQ	-0.3 [-0.8; 0.1]	0.7 [0.6; 1.3]	0.0717
**FXYD domain-containing ion transport regulator 5**	**Q96DB9**	FXYD5	-0.9 [-1.1; -0.2]	0.4 [0.3; 0.8]	0.0720
**Cytoskeleton-associated protein 4**	**Q07065**	CKAP4	4.5 [3.4; 4.7]	5.5 [5.3; 6.9]	0.0842
**Interleukin-10**	**P22301**	IL10	2.5 [1.8; 2.8]	1.8 [1.2; 2.6]	0.1001
**Protein-arginine deiminase type-2**	**Q9Y2J8**	PADI2	0.0 [-0.4; 1.6]	1.7 [0.6; 2.0]	0.1043
**C-type lectin domain family 4 member D**	**Q8WXI8**	CLEC4D	1.5 [1.0; 2.4]	2.9 [2.6; 4.1]	0.1298
**Fc receptor-like protein 3**	**Q96P31**	FCRL3	0.7 [0.1; 0.8]	1.0 [0.4; 1.7]	0.1388
**Transcription factor Jun**	**P05412**	JUN	0.7 [-1.0; 1.0]	0.5 [0.3; 2.2]	0.1454
**Lymphocyte activation gene 3 protein**	**P18627**	LAG3	1.3 [0.5; 1.3]	1.3 [0.5; 1.3]	0.1686
**C-type lectin domain family 6 member A**	**Q6EIG7**	CLEC6A	1.0 [0.9; 1.3]	2.0 [1.3; 2.3]	0.1711
**Amphiregulin**	**P15514**	AREG	2.0 [1.6; 2.1]	2.3 [2.2; 2.4]	0.1752
**Natural killer cells antigen CD94**	**Q13241**	KLRD1	5.8 [5.4; 5.8]	6.1 [5.5; 6.6]	0.1755
**Contactin-associated protein-like 2**	**Q9UHC6**	CNTNAP2	0.6 [0.5; 0.8]	0.9 [0.6; 1.1]	0.2607
**Aryl hydrocarbon receptor nuclear translocator**	**P27540**	ARNT	-0.4 [-0.4; 0.5]	0.6 [-0.4; 0.8]	0.2827
**Tryptase alpha/beta-1**	**Q15661**	TPSAB1	4.8 [4.2; 5.2]	5.1 [4.5; 5.8]	0.3291
**Corneodesmosin**	**Q15517**	CDSN	3.4 [2.8; 6.2]	2.5 [2.4; 3.3]	0.3439
**Fc receptor-like protein 6**	**Q6DN72**	FCRL6	2.0 [1.5; 2.2]	1.9 [1.4; 2.1]	0.3449
**Protein FAM3B**	**P58499**	FAM3B	3.0 [1.9; 3.2]	3.0 [1.9; 3.4]	0.3538
**11-beta-hydroxysteroid dehydrogenase 1**	**P28845**	HSD11B1	2.2 [1.4; 2.3]	2.3 [1.5; 2.5]	0.3778
**Lysosome-associated membrane glycoprotein 3**	**Q9UQV4**	LAMP3	3.2 [2.7; 4.6]	3.3 [2.6; 4.9]	0.3809
**Integrin beta-6**	**P18564**	ITGB6	2.0 [1.9; 2.1]	2.1 [2.0; 2.3]	0.4369
**Importin subunit alpha-5**	**P52294**	KPNA1	0.4 [-1.8; 0.8]	0.2 [-0.2; 0.9]	0.4532
**Interferon lambda receptor 1**	**Q8IU57**	IFNLR1	1.8 [1.7; 1.9]	1.7 [1.7; 1.9]	0.5015
**C-type lectin domain family 4 member C**	**Q8WTT0**	CLEC4C	3.4 [1.9; 3.4]	3.2 [2.0; 4.0]	0.5037
**Neurotrophin-4**	**P34130**	NTF4	0.8 [0.6; 3.5]	0.9 [0.6; 3.1]	0.6519
**Keratin, type I cytoskeletal 19**	**P08727**	KRT19	1.5 [1.3; 2.3]	1.9 [1.0; 2.6]	0.6551
**Discoidin, CUB and LCCL domain-containing protein 2**	**Q96PD2**	DCBLD2	7.1 [7.1; 7.4]	7.2 [7.1; 7.2]	0.6861
**Eukaryotic translation initiation factor 5A-1**	**P63241**	EIF5A	-0.1 [-0.2; 0.2]	-0.0 [-0.5; 0.1]	0.7124

**CD83 antigen**	**Q01151**	CD83	1.5 [1.2; 2.0]	1.8 [1.3; 1.8]	0.7209
**C-type lectin domain family 4 member G**	**Q6UXB4**	CLEC4G	2.4 [2.0; 2.6]	2.5 [2.0; 2.5]	0.7648
**Interleukin-6**	**P05231**	IL6	1.4 [0.8; 2.2]	1.2 [1.0; 2.1]	0.8789
**Allergin-1**	**Q7Z6M3**	MILR1	2.2 [1.7; 2.9]	2.0 [1.7; 3.1]	0.8843
**Beta-galactosidase**	**P16278**	GLB1	-0.3 [-1.4; 0.07]	-0.4 [-0.9; -0.3]	0.9509
