# Supplementary material for: N-Acetylcysteine Attenuates Oxidative Stress and Preserves Red Blood Cell Quality During Whole Blood Storage
Source: Antioxidants (Basel). 2026 Jul 8;15(7):858. doi: 10.3390/antiox15070858 (PMC13404811; doi:10.3390/antiox15070858)
Supplement: Supplementary file 1 [file antioxidants-15-00858-s001.zip › figure S1.pptx]

## Slide 1
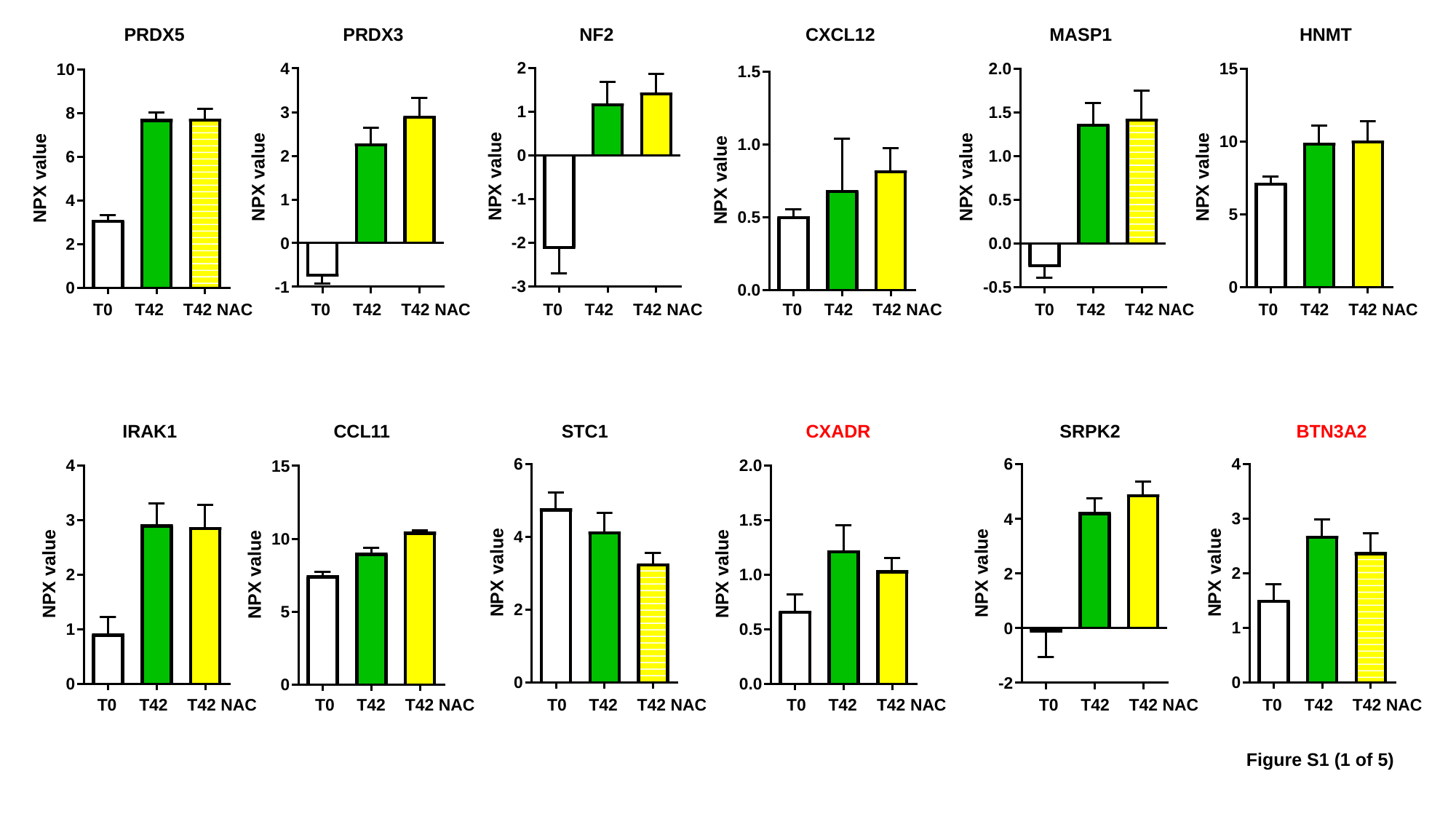

PRDX5
PRDX3
NF2
CXCL12
MASP1
HNMT
T0
T42
T42 NAC
T0
T42
T42 NAC
T0
T42
T42 NAC
T0
T42
T42 NAC
T0
T42
T42 NAC
T0
T42
T42 NAC
IRAK1
CCL11
STC1
CXADR
SRPK2
BTN3A2
T0
T42
T42 NAC
T0
T42
T42 NAC
T0
T42
T42 NAC
T0
T42
T42 NAC
T0
T42
T42 NAC
T0
T42
T42 NAC
Figure S1 (1 of 5)

## Slide 2
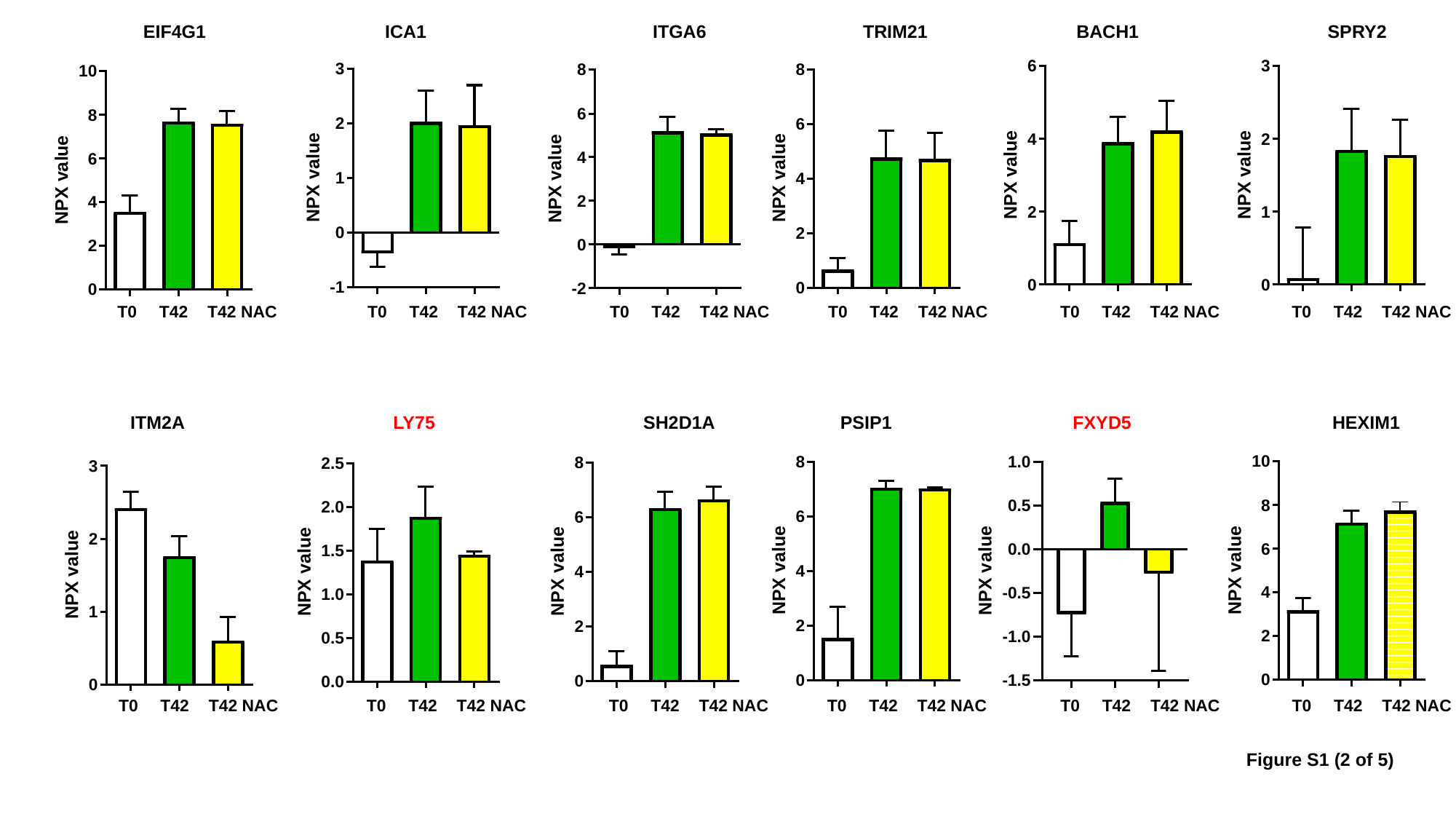

EIF4G1
ICA1
ITGA6
TRIM21
BACH1
SPRY2
T0
T42
T42 NAC
T0
T42
T42 NAC
T0
T42
T42 NAC
T0
T42
T42 NAC
T0
T42
T42 NAC
T0
T42
T42 NAC
ITM2A
LY75
SH2D1A
PSIP1
FXYD5
HEXIM1
T0
T42
T42 NAC
T0
T42
T42 NAC
T0
T42
T42 NAC
T0
T42
T42 NAC
T0
T42
T42 NAC
T0
T42
T42 NAC
Figure S1 (2 of 5)

## Slide 3
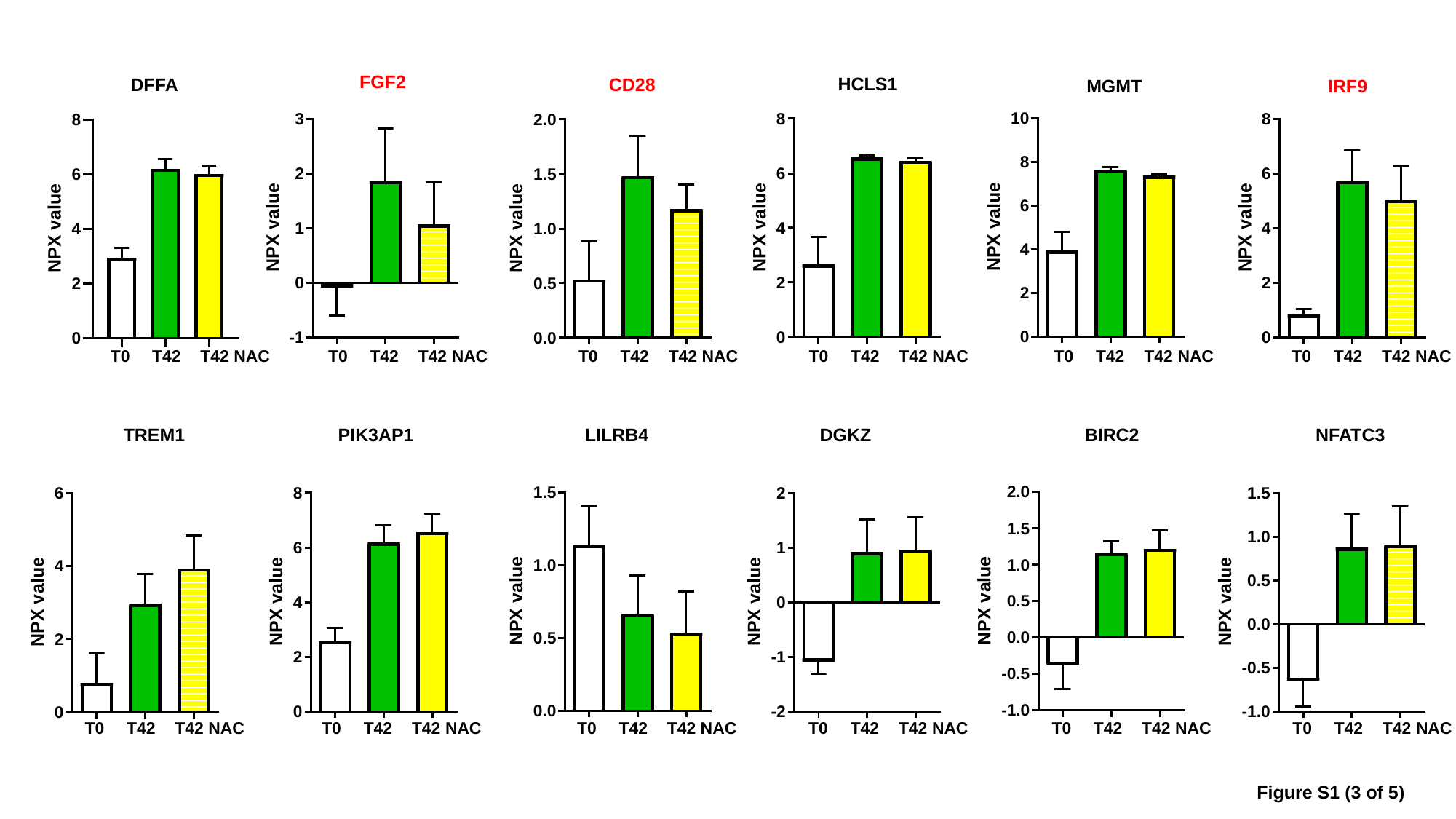

FGF2
HCLS1
DFFA
CD28
IRF9
MGMT
T0
T42
T42 NAC
T0
T42
T42 NAC
T0
T42
T42 NAC
T0
T42
T42 NAC
T0
T42
T42 NAC
T0
T42
T42 NAC
TREM1
PIK3AP1
LILRB4
DGKZ
BIRC2
NFATC3
T0
T42
T42 NAC
T0
T42
T42 NAC
T0
T42
T42 NAC
T0
T42
T42 NAC
T0
T42
T42 NAC
T0
T42
T42 NAC
Figure S1 (3 of 5)

## Slide 4
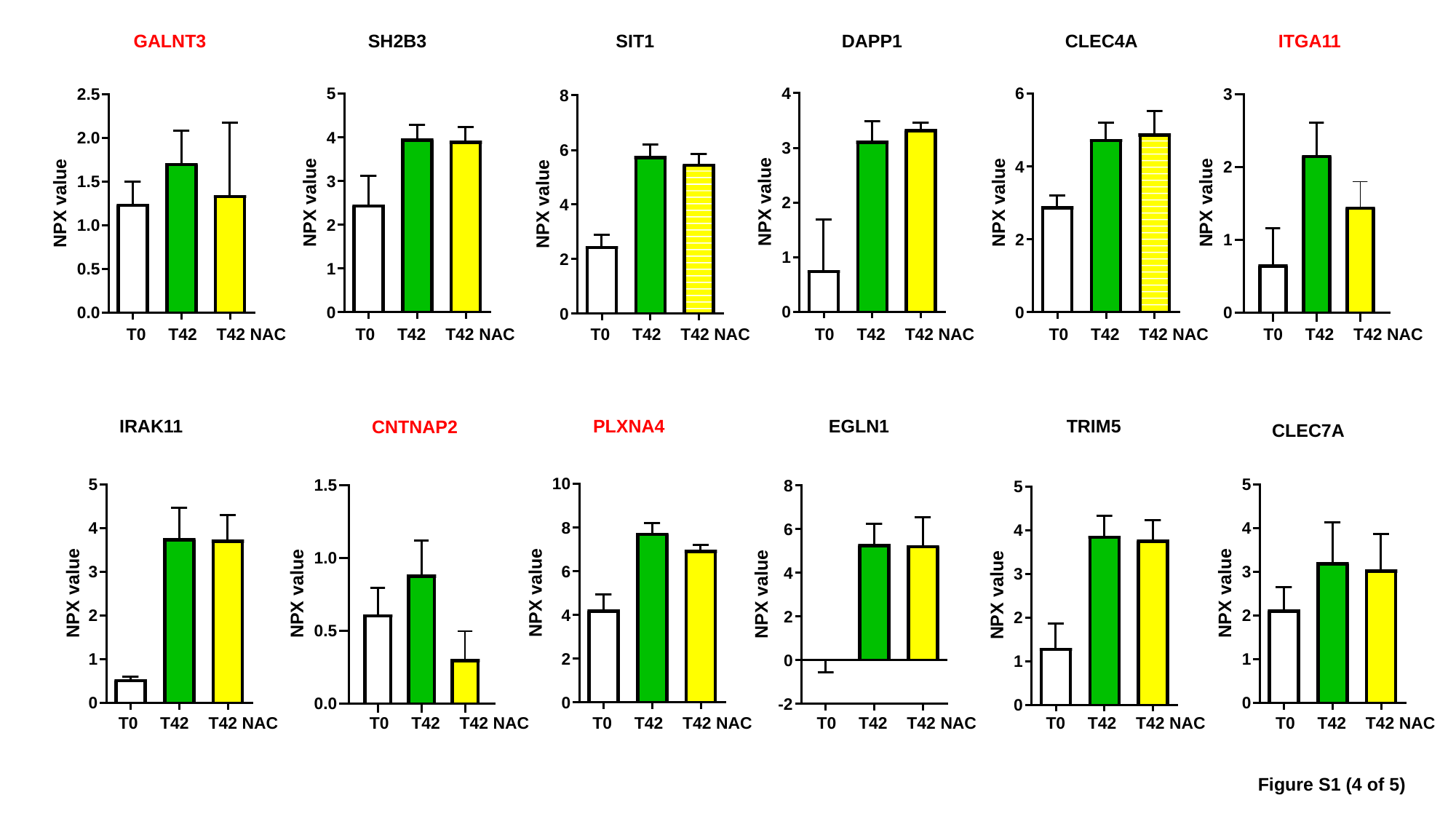

GALNT3
SH2B3
SIT1
DAPP1
CLEC4A
ITGA11
T0
T42
T42 NAC
T0
T42
T42 NAC
T0
T42
T42 NAC
T0
T42
T42 NAC
T0
T42
T42 NAC
T0
T42
T42 NAC
EGLN1
TRIM5
IRAK11
PLXNA4
CNTNAP2
CLEC7A
T0
T42
T42 NAC
T0
T42
T42 NAC
T0
T42
T42 NAC
T0
T42
T42 NAC
T0
T42
T42 NAC
T0
T42
T42 NAC
Figure S1 (4 of 5)

## Slide 5
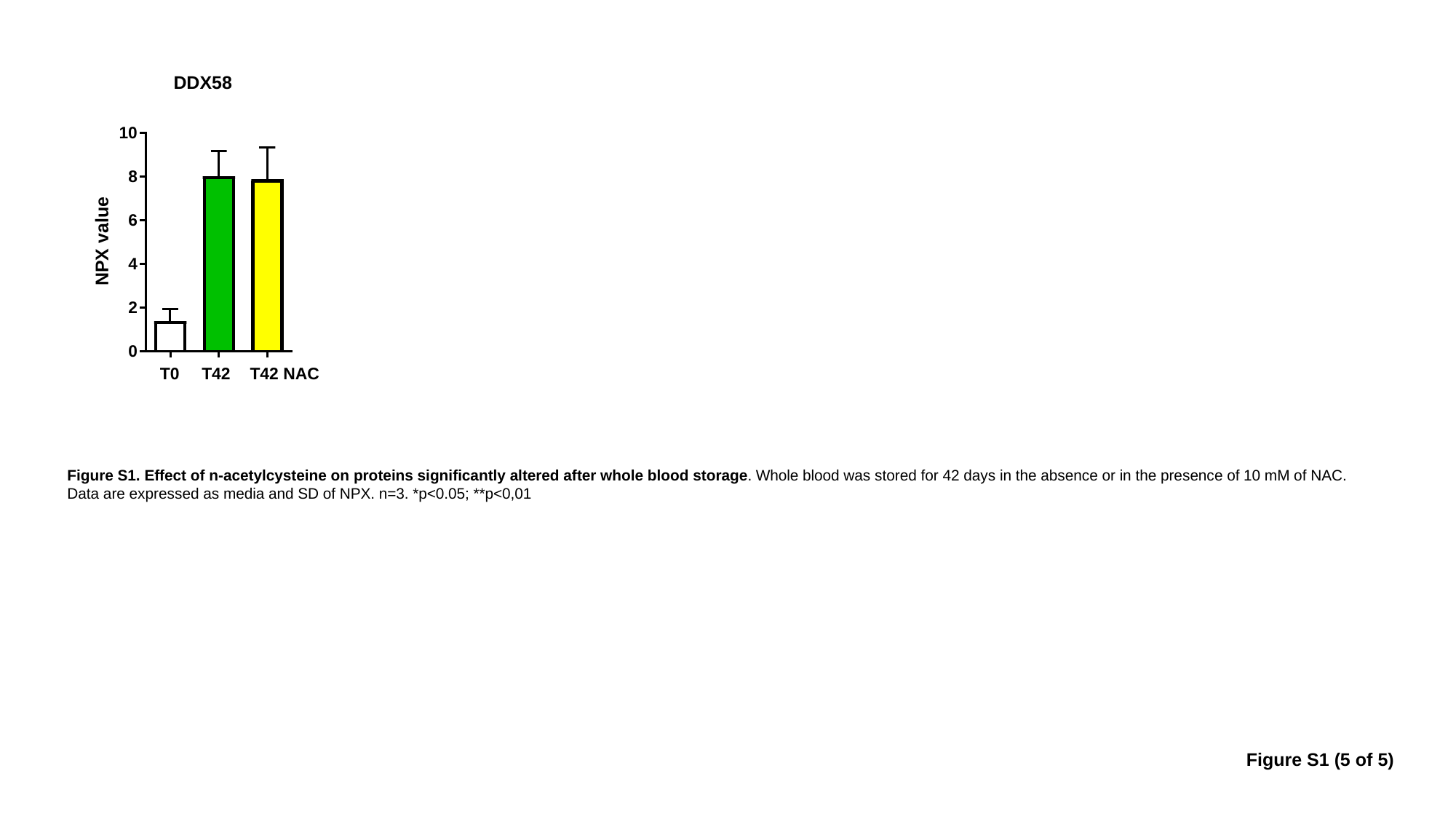

DDX58
T0
T42
T42 NAC
Figure S1. Effect of n-acetylcysteine on proteins significantly altered after whole blood storage. Whole blood was stored for 42 days in the absence or in the presence of 10 mM of NAC. Data are expressed as media and SD of NPX. n=3. *p<0.05; **p<0,01
Figure S1 (5 of 5)
