# Supplementary material for: N-Acetylcysteine Attenuates Oxidative Stress and Preserves Red Blood Cell Quality During Whole Blood Storage
Source: Antioxidants (Basel). 2026 Jul 8;15(7):858. doi: 10.3390/antiox15070858 (PMC13404811; doi:10.3390/antiox15070858)
Supplement: Supplementary file 1 [file antioxidants-15-00858-s001.zip › figure S2.pptx]

## Slide 1
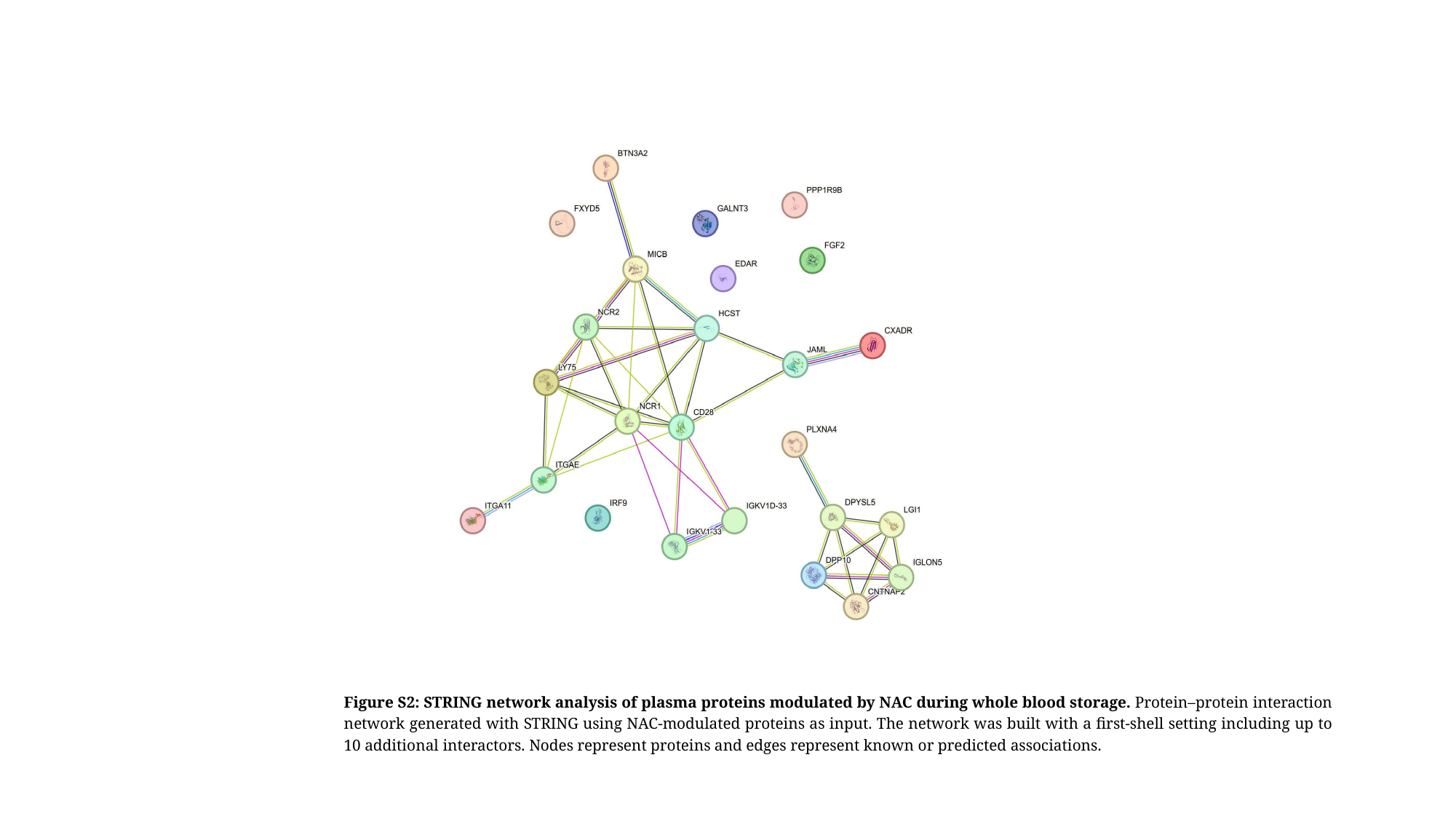

Figure S2: STRING network analysis of plasma proteins modulated by NAC during whole blood storage. Protein–protein interaction network generated with STRING using NAC-modulated proteins as input. The network was built with a first-shell setting including up to 10 additional interactors. Nodes represent proteins and edges represent known or predicted associations.
